# Prospective international multicenter observational study of Novosyn® Quick for skin closures in adults and children (SKINNOQ)

**DOI:** 10.1186/s12893-019-0506-8

**Published:** 2019-05-02

**Authors:** Stefan Gfroerer, Petra Baumann, Anne-Kathrin Schwalbach, Alexandre Smirnoff

**Affiliations:** 1 Department of Paediatric Surgery and Paediatric Urology, University Hospital Frankfurt, Theodor-Stern-Kai 7, 60590 Frankfurt am Main, Germany; 2 Aesculap AG, Medical Scientific Affairs, 78532 Tuttlingen, Germany; 3 Service de chirurgie viscérale, Hôpital Saint-Louis, La Rochelle, France

**Keywords:** Skin closure, Fast-absorbable braided suture, Paediatric

## Abstract

**Background:**

This clinical trial evaluated the performance of a newly released fast-absorbable braided synthetic suture (Novosyn® Quick) in adults and paediatric patients undergoing elective skin closures.

**Methods:**

This was a prospective international multicentre observational study. Two centres enrolled 100 patients, of which 50 were adults (visceral surgery, France) and 50 were paediatric patients (paediatric surgery, Germany). Surgeons used a 5-point Likert scale to assess handling characteristics of the suture. Patients and professionals used the Patient-Observer-Scar-Assessment-Scale (POSAS) to rate scar quality. Adverse events were monitored until 3 months postoperatively.

**Results:**

Handling characteristics of Novosyn® Quick were in median rated very good by both general surgeons and paediatric surgeons. Patient components of POSAS (six questions; ten-level Likert scale; best possible score six) scored in median (range) 8.5 (6–28) in the paediatric group versus 12 (6–38) in the adult group, *P* = 0.01. Patients` overall opinions of POSAS were similar in both groups [mean (SD), 1.86 (0.99) in the paediatric group versus 2.08 (1.35) in the adult group, *P* = 0.3536]. Observer component of POSAS (six parameters; ten-level numeric rating scale, best possible score six) scored comparably in both groups [median (range), 8 (6–29) in the paediatric group versus 10 (6–28) in the adult group, *P* = 0.1403]. Observers overall opinion of POSAS favoured the paediatric patients group [mean (SD), 1.48 (0.61) versus 1.92 (1.06) in the adult group, *P* = 0.0131]. Adverse events in relation to wound healing were not observed in both patient groups.

**Conclusions:**

Our findings indicate, that Novosyn*®* Quick is safe and reliable for skin closure in adults and paediatric patients and can be regarded as a viable alternative to Vicryl*®* Rapide.

**Trial registration:**

This trial was registered prospectively with ClinicalTrials.gov under the registration number NCT02680886 on 5 February 2016.

The trial was approved by the Institutional Review Boards of both study locations (France: CCTIRS N° 16–103 and CNIL:MMS/CWR/AR163920; Germany: 398/15).

**Electronic supplementary material:**

The online version of this article (10.1186/s12893-019-0506-8) contains supplementary material, which is available to authorized users.

## Background

Suture material for skin closure should be easy to handle, provide sufficient strength during the proliferative phase of wound healing, facilitate the best possible aesthetic result and should not cause more than an expected, normal local inflammatory reaction. Traditionally non-absorbable monofilament sutures have been used for skin closure in adults and children. However, removal of the suture potentially causes discomfort for the patient and might be time-consuming. With the use of absorbable sutures pain and anxiety associated with suture removal are avoided. Time saved on the patient reduces costs and minimises burden on medical staff. Polyglactin 910 (Vicryl®), as an absorbable synthetic suture material, is frequently used for wound closure. This product was introduced to the market in 1974. A fast-absorbable suture (Vicryl® Rapide) was approved for the market in 1987. Vicryl® Rapide gives wound support up to 14 days post-implantation and has a total absorption time of 42 days. Several publications are available decribing the use of Vicryl® Rapide for skin closures in children and in adults [1–17]. Vicryl® Rapide can be regarded as a market reference for fast-absorbable, braided suture material.

Recently a competing product, Novosyn® Quick, was released. The product has marketing authorization and CE-mark since 21 May 2015. Novosyn® Quick is a braided multifilamentous copolymer of 90% glycolide and 10% L-lactide. The threads are coated with glycolide, L-lactide and calcium stearate and are gamma-irradiated. 5 days post-implantation the suture has lost 50% of its strength and 14 days post-implantation 100%. At approximately 42 days post implantation absorption of Novosyn® Quick is completed. In this prospective clinical observational study we aimed to collect data on the performance of Novosyn® Quick applied during routine clinical practice in adults and paediatric patients. The study was part of an obligatory post-marketing surveillance analysis. Various parameters were used to assess the safety and efficacy of this suture material for skin closure.

## Methods

### Study design and participants

**Fig. 1:**
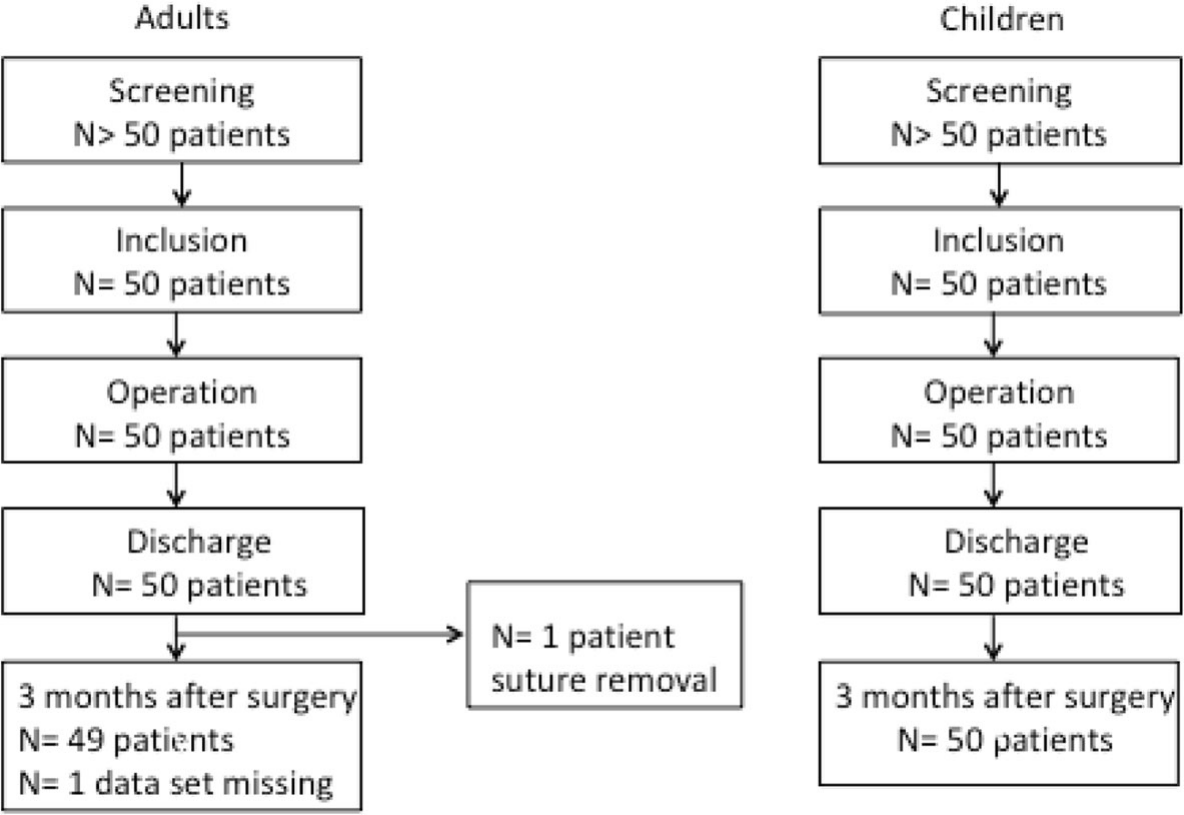
Adult patients and paediatric patients flow diagram

We designed a prospective, non-interventional, international, multi-center single-arm cohort study to assess intraoperative handling, wound healing outcomes and postoperative patients` satisfaction of Novosyn® Quick for skin closures in adults and paediatric patients. The trial was registered with ClinicalTrials.gov under the registration number NCT02680886; the trial registration was on February 5, 2016. A total of 148 patients were screened, finally 100 patients were enrolled and assigned to the adults (*N* = 50) or the paediatric (N = 50) patient group. One study center was located in France (visceral surgery), and one in Germany (paediatric surgery). The flow chart of patient screening, inclusion, operation and follow-up is shown in Fig. 1. The inclusion criteria were as follows: paediatric and adult patients undergoing routine skin closure of linear, minimally contaminated incisions of the trunk or extremities and a written informed consent. Exclusion criteria were emergency surgery, facial surgery, contaminated wounds, nonlinear shape and consumption of drugs that might have affected wound healing (e.g. immunosuppressive medication). Patients were treated according to the local standards. Novosyn® Quick was regularly used for skin closures by all participating surgeons in both centers for longer than 3 months prior to inclusion of the first patient. The suture was used to stitch the dermal wound. Interrupted transcutaneous or continuous intradermal suture technique was applied as chosen by the surgeon. The total study duration for each patient was 3 months ±2 weeks. There were four visitations for evaluation of the patient: screening, operation, discharge and follow-up at 3 months ±2 weeks postoperatively. Novosyn® Quick suture was applied in routine clinical practice and according to its instructions for use. The data were collected either in a paper-based case report form (CRF) or in an online database, as preferred by the participating investigator (Additional file 1).

### Outcomes

**Fig. 2:**
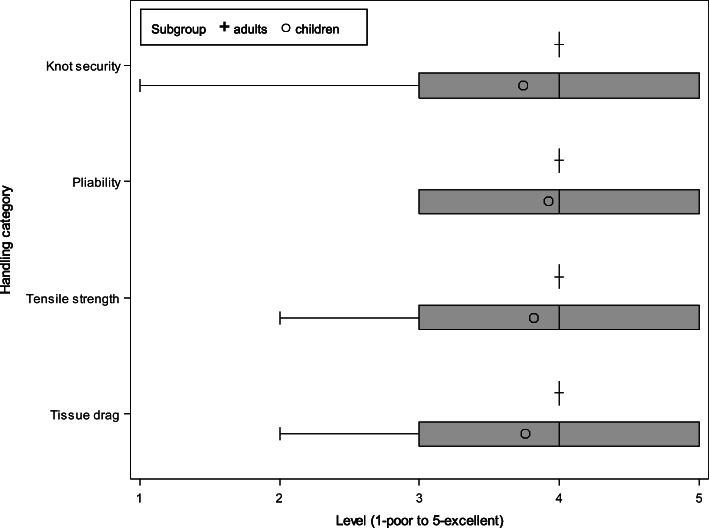
Box plot of intraoperative handling characteristics of Novosyn® Quick

As a parameter of efficacy the suture was assessed for its intraoperative handling characteristics by all surgeons (Fig. 2). Surgeons of the study centre in France and Germany completed a questionnaire after each skin closure of an enrolled patient. Categories of intraoperative handling assessment were knot security, tensile strength, tissue drag and pliability of the suture. Each category was rated on a five-point Likert-type scale as follows: excellent, very good, good, satisfied and poor. Additionally, intraoperative complications of the suture (thread rupture, knots in the thread, bended thread, defect in needle-thread attachment, and others) were recorded.

Further, efficacy was evaluated based on results of the scar quality assessment 3 months after surgery using the Patient-Observer-Scar-Assessment-Scale (POSAS). POSAS is an internationally validated measure for the evaluation of scar quality [18–20]. It is a comprehensive scale that is designed for the evaluation of all types of scars by professionals and patients (https://www.posas.org/). Medical staff used the observer component of the POSAS to evaluate the scar quality on a ten-level numeric rating scale. The observer component of the POSAS included following criteria (original notices extracted from https://www.posas.org/): 1) vascularity (presence of vessels in scar tissue assessed by the amount of redness, tested by the amount of blood return after blanching with a piece of Plexiglas; 2) pigmentation (brownish coloration of the scar by pigmentation; apply transparent Plexiglas to the skin with moderate pressure to eliminate the effect of vascularity; 3) thickness (average distance between the subcuticular-dermal border and the epidermal surface of the scar); 4) relief (the extent to which surface irregularities are present, preferably compared with adjacent normal skin); 5) pliability (suppleness of the scar tested by wrinkling the scar between the thumb and index finger); 6) surface area (surface area of the scar in relation to the original wound area). The patient component gives the POSAS an important extra dimension, because the patient’s opinion is mandatory for a complete scar evaluation. The patient component of POSAS included six questions, which had to be answered on a 10-point Likert scale (like normal skin; very different to normal skin). For patients, that were unable to assess their wounds (infants, children), parents were asked to answer the questionnaire.

Criteria for safety evaluation were postopersative adverse events, which included following complications until 3 months after surgery: surgical wound dehiscence, (defined as the separation of the margins of a closed surgical cutaneous incision, with or without exposure or protrusion of underlying tissue, occurring at single or multiple regions, or involving the full length of the incision and affecting some or all tissue layers; the dehisced incision may, or may not, display clinical signs and symptoms of infection), surgical site infection (defined as infection of the skin and subcutaneous tissue of the incision occuring within 30 days after the operative procedure), tissue reaction, allergic reaction, inflammation, and the need for suture removal due to incomplete- or non-absorbed suture. Serious adverse events were specified as follows: required or prolonged hospitalization, need for surgery to prevent permanent impairment of body structure or function, permanent impairment of body structure or function, life threatening event or death. Wound healing was evaluated both at discharge from hospital and 3 months (± 2 weeks) postoperatively.

### Statistical methods and sample size

All patients who had a surgical intervention using the product under investigation without any protocol violation were included in the per-protocol analysis. A sample size of 100 patients was considered to be appropriate to detect absence of excessive adverse events. In addition the sample size was determined based on the number of patients, which could be recruited in a small multi-centric study within a reasonable period of time. The results from the present post-marketing clinical follow-up were analysed descriptively and compared with corresponding data from the literature. If the data of this study correspond to published data of Vicryl® Rapide, the suture material Novosyn® Quick was regarded as safe. For comparison of our study results with corresponding ranges from the literature, 95% confidence intervals (Agresti-Coull method) were applied. Confidence intervals allowed establishing ranges for each analysed parameter. Statistical analysis was performed using SAS software version 9.4 (SAS Institute Inc., Cary, NC, USA). Variables with metric or ordinal scale were summarised with number of patients (N), minimum (Min), maximum (Max), median, mean, and standard deviation (SD). Categorical variables were summarised by category to absolute (N) and relative (%) frequency with missing values building an own category. Missing data were analysed as such and were not replaced by estimates. For statistical comparison the t-test was used.

### Ethical considerations

No study-related investigations or interventions were carried out on the patient. Study was performed under daily clinical practice. The product under investigation was used according to approved instructions for use. The patient was treated in a way the physician considered it to be the most appropriate. The study was presented to the local Ethics Committee of each participating center. Before initiation of the study, protocol, consent form, and CRF were approved by each local institutional review board (France IRB No.: CCTIRS N° 16–103; CNIL:MMS/CWR/AR163920; Germany IRB No.: 398/15).

## Results

### Initiation and completion of patient recruitment

The first adult patient was included in this study on January 18, 2017. Recruitment of 50 patients was completed on March 21, 2017. The first paediatric patient was included on March 22, 2016. Recruitment of 50 paediatric patients was completed on September 22, 2017. Last patient visitation in the adult and paediatric group was June 30, 2017 and December 28, 2017 respectively.

### Visitations

Of fifty adult patients that were included, forty-nine had undergone all four visitations. One adult patient had undergone early postoperative suture removal due to a subcutaneous hematoma. For this patient data of the 3-months scar assessment were not available (Fig. 1). Of fifty paediatric patients all had undergone all four study related visitations.

### Patient demographics and other baseline characteristics

Age, gender, weight, height and Body Mass Index (BMI) of adult and paediatric patients are listed in Table 1. In total 32 men and 18 women were included in the study. The median (range) BMI of adults was 27.19 (19.27–39.33) kg/m^2^. The adult patients` median (range) age was 62 (30–87) years. One adult patient was African and 49 were Caucasian. In total 32 boys and 18 girls were included in the study. The median (range) age was 2.92 years (1 month-13 years). The paediatric population was subdivided into 3 groups: 0–1 year (*N* = 19), 1 to 5 years (*N* = 17) and older than 5 years (*N* = 14). Demographic data related to paediatric age groups are listed in Table 1. All paediatric patients were Caucasian.

**Table 1:** Demographic data for 50 adult and 50 paediatric patients (children) undergoing cutaneous wound closure with Novosyn® Quick

		Adults		Children	
		N	Median (range)	N	Median (range)
Age (years)		50	62 (30–87)	50	2.92 (0.08–13)
Weight (kg)		50	82.5 (53–131)	50	13 (2.4–60)
Age category	Gender			3	3.93 (2.98–3.93)
0 to 1 years	Female				
	Male			16	3.78 (2.4–7.5)
1 to 5 years	Female			2	13.65 (13–14.3)
	Male			15	14 (10–19)
> 5 years	Female	18	70.5 (53–111)	3	26.5 (26–41)
	Male	32	85 (62–131)	11	26 (18–60)
Height (cm)		50	172 (156–193)	50	95 (41–156)
Age category	Gender			3	50 (50)
0 to 1 years	Female				
	Male			16	52 (41–69)
1 to 5 years	Female			2	95.5 (95–96)
	Male			15	96 (76–110)
> 5 years	Female	18	165 (156–173)	3	140 (134–156)
	Male	32	174 (160–193)	11	133 (105–155)
BMI (kg/m^2^)		50	27.19 (19.27–39.33)	50	15.16 (11.2–28.93)
Age category				19	15.31 (11.2–17.27)
0 to 1 years					
1 to 5 years				17	14.4 (12.35–19.04)
> 5 years		50	27.19 (19.27–39.33)	14	15.23 (12.71–28.93)

*BMI* Body mass index, *N* Numbers

### Surgery details

One visceral surgeon in the centre in France and seven paediatric surgeons in the centre in Germany performed skin closures of enrolled patients. Reasons for surgery in the adult and the paediatric group are listed in Table 2. Inguinal hernia repair was the most frequent reason for surgery in the adult group followed by ventral hernia repair, cholecystectomy, colon tumour surgery, umbilical hernia repair, sigmoid diverticulitis, hiatal hernia operation, lipoma and parietal endometriosis surgery. Main reason for surgery in children was inguinal hernia repair (*N* = 41), followed by orchidopexy (*N* = 6), skin excision (*N* = 2) and umbilical hernia repair (*N* = 1). Skin incisions were performed in abdominal, umbilical or inguinal regions of the body (Table 3). The median number of threads used for skin closure was 1 in both groups. Skin closure was predominantly performed using an interrupted suture technique (*N* = 45) and less often using a continuous suture technique (*N* = 5) in the adult group as opposed to the paediatric group where 98% of the sutures were performed using a continuous technique. Median length of incision was longer in adult patients compared with paediatric patients [median (range) 5.5 (2–16) versus 2.5 (1.5–4.0) cm, *P* < 0.0001]. Mean numbers of threads used per skin closure were higher in the adult patient group compared with the paediatric patient group [mean (SD), 1.50 (0.61) versus 1.10 (0.36) N, *P* = 0.0001].

**Table 2:** Reasons for elective surgery of 50 adult and 50 paediatric patients (children) that underwent cutaneous wound closure with Novosyn® Quick

Reason for surgery	Adults		Children	
	N	%	N	%
Superficial surgery				
Inguinal hernia	16	32%	41	82%
Ventral hernia	9	18%		
Orchiopexy			6	12%
Umbilical hernia	3	6%	1	2%
Lipoma	1	2%		
Skin excision			2	4%
Deep surgery				
Sigmoid resection	2	4%		
Hiatal hernia	1	2%		
Colon tumor	7	14%		
Cholelithiasis	10	20%		
Parietal endometriosa	1	2%		

*N* Numbers, % frequency, *N* 13 (26%) patients of the adults group received a perioperative single-shot antibiotic prophylaxis (Cefuroxim), *N* = 9 (18%) patients of the adults group received a peri−/ postoperative antibiotic treatment between 3 and 9 days (Cefuroxim, Metronidazole); none of the paediatric patients received a peri- or postoperative antibiotic treatment

**Table 3:** Localisation of incision, suture technique and suture characteristics in 50 adult and 50 paediatric patients (children) undergoing cutaneous wound closure with Novosyn® Quick

		Adults		Children	
		N	%	N	%
Incision site					
Body - frontal					
Inguinal		3	6%	47	94%
Abdominal		35	70%		
Umbilical		12	24%	1	2%
Suture line					
Transversal		40	80%	48	96%
Longitudinal		10	20%		
Body - dorsal					
Lumbo-sacral				1	2%
Left infrascapular				1	2%
Suture line					
Oblique				2	4%
Suture technique Skin					
Continuous		5	10%	49	98%
Interrupted		45	90%	1	2%
Subcutaneous tissue					
Sutures applied		41	82%	42	84%
USP Size	Needle characteristic				
3/0	DSMP	48	96%		
4/0	DSMP	2	4%		
5/0	DSMP			50	100%
Number of threads used					
1		27	54%	46	92%
2		22	44%	3	6%
3				1	2%
4		1	2%		
Number of threads used, median (range)		1 (1–4)		1 (1–3)	
Length of incision, median (range), (cm)		5.5 (2–16)		2.5 (1.5–4)	

*N* Numbers, *%* Frequency, *USP* United States Pharmacopeia, *DSMP* Needle angel of curvature (D) - 3/8 circle; body type (S) - cutting needle; point type (MP) - micro-point precision

### Intraoperative handling of suture material

Median level of surgeons` suture handling assessment in both adult and paediatric group was 4 (very good) (Fig. 2). Surgeons of the adult group rated all handling categories with 4 (range 4–4). In the paediatric patient group ratings were more divers, which is reflected in a wider range of median rating for each category.

### Adverse events and adverse device effects

In the paediatric patient group the following intraoperative adverse events were reported: one wound needed twice intraoperative resuturing due to knot openings; three threads ruptured (size 5/0); one rupture was combined with accidental knots in the thread (Table 4). In both patient groups no adverse events in relation to wound healing over the study period of 3 months were discovered. In the adult group one suture had to be removed for a subcutaneous hematoma to be evacuated during early postoperative in-patient treatment.

**Table 4:** Intraoperative and postoperative adverse events (AE) and adverse device effects (ADE) in 50 adult and 50 paediatric patients undergoing cutaneous wound closure with Novosyn® Quick

	Specification	Adults		Children	
		N	%	N	%
Intraoperative AE and ADE					
No	–	50	100%	46	92%
Yes	Thread rupture	–		2	4%
	Knot in the thread	–		1	2%
	Knot opening, re-do suture	–		1	2%
Postoperative in-patient AE					
No	–	49	98%	50	100%
Yes	Subcutaneous haematoma	1	2%	–	
Postoperative out-patient AE					
No	–	50	100%	50	100%

*N* Numbers, % Frequency

### Length of hospital stay

Mean length of hospital stay in the adult group was longer than that in the paediatric patients group [mean (SD) 3.40 (4.86) versus 0.56 (0.54) days respectively, *P* < 0.0001].

### Postoperative scar assessment using POSAS

At 3 months ±10 days postoperatively professional medical staff and patients/ parents assessed the scar quality using the appropriate component of the POSAS questionnaire (Table 5). POSAS observer total score did not differ between both groups [mean (range), 10.04 (6–29) in the paediatric group versus 11.65 (6–28) in the adult group, *P* = 0.1403] however observers` overall opinion favoured the paediatric patients group [mean (range), 1.48 (1–3) versus 1.92 (1–5) in the adult group, *P* = 0.0131]. POSAS patient overall opinions were similar in both groups [mean (range), 1.86 (1–5) in the paediatric group versus 2.08 (1–6) in the adult group, *P* = 0.3536] while POSAS total score of patient component scored better in the paediatric group [mean (range), 10.6 (6–28) versus 13.94 (6–38) in the adult group, *P* = 0.0102].

**Table 5:** Patient-Observer-Scar-Assessment-Scale (POSAS) of 50 adult and 50 paediatric patients (children) at 3 months follow-up after cutaneous wound closure with Novosyn® Quick

	Adults (*N* = 49^a^)	Children (N = 50)	*P*
POSAS Observer Parameter (score 1–10, best-worst)			
Vascularity	1.82 (1–4)	1.76 (1–5)	0.7357
Pigmentation	2.29 (1–5)	2.04 (1–7)	0.3494
Thickness	2.00 (1–5)	1.68 (1–9)	0.2049
Relief	1.96 (1–5)	1.66 (1–7)	0.2202
Pliability	1.82 (1–5)	1.42 (1–3)	**0.0340**
Surface Area	1.78 (1–5)	1.48 (1–5)	0.1489
Overall opinion	1.92 (1–5)	1.48 (1–3)	**0.0131**
POSAS total score (6–60, best-worst)	11.65 (6–28)	10.04 (6–29)	0.1403
POSAS Patient Question (score 1–10, best-worst)			
Has the scar been painful the past few weeks?	1.78 (1–8)	1.04 (1–3)	**0.0004**
Has the scar been itching the past few weeks?	2.06 (1–8)	1.00 (1–1)	**0.0002**
Is the color different from the color of your normal skin at present?	3.22 (1–9)	3.54 (1–10)	0.5140
Is the stiffness of the scar different from your normal skin at present?	2.45 (1–7)	1.80 (1–10)	0.0551
Is the thickness of the scar different from your normal skin at present?	2.37 (1–7)	1.56 (1–5)	**0.0044**
Is your scar more irregular than your normal skin at present?	2.06 (1–8)	1.66 (1–5)	0.1258
What is your overall opinion of the scar compared to normal skin?	2.08 (1–6)	1.86 (1–5)	0.3536
POSAS total score (6–60, best-worst)	13.94 (6–38)	10.6 (6–28)	**0.0102**

data are mean (range); N = numbers; bold numbers represent *P* values < 0.05; ^a^49 of 50 patients in the adults group underwent scar assessment at 3 months follow-up; for 1 patient scar assessment data were missing due to postoperative suture removal because of a subcutaneous haematoma

**Fig. 3:**
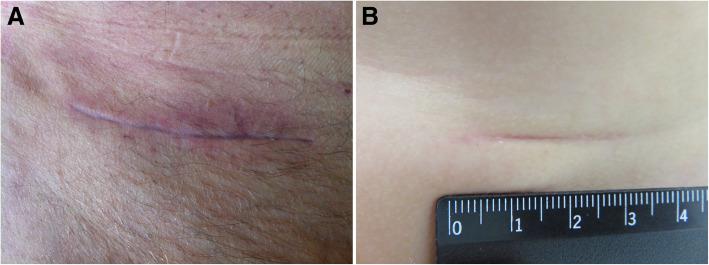
**a** Exemplary photodocumentation of an adult patient´ scar (62 year old patient, inguinal hernia repair) and **b** paediatric patient´ scar (5½ months old infant, inguinal hernia repair) at 3 months postoperative assessment

Figure 3 a + b display an exemplary scar in the adult and paediatric patient group at 3 months follow-up.

## Discussion

This was a postmarketing surveillance study and is, to our knowledge, the first study evaluating the safety and performance of the newly introduced fast-absorbable suture (Novosyn® Quick) for cutaneous wound closures in adults and paediatric patients. In this study wound healing complications in causal relationship to cutaneous Novosyn® Quick suture application was 0% in the adult and the paediatric patient group. Levels of suture handling characteristics were rated in median very good by surgeons of both adult and paediatric patient group. Mean overall opinion according to the Patient-Observer-Scar-Assessment-Scale (POSAS) at 3 months postoperatively ranged between 1.48 and 2.08 (rating scale 1–10; normal; very different) indicating, that the use of Novosyn® Quick permits a very good to excellent scar quality in adults and paediatric patients.

In 1987 Vicryl® Rapide, a fast-absorbable suture material, was introduced to the market. The composition of this suture material was identical to standard Vicryl®, but absorption was much faster due to an additional gamma irradiation. Several publications studied Vicryl® Rapide for skin closure in children and in adults [1–17]. Publications confirmed, that fast-absorbable Vicryl® Rapide for closures of cutaneous wounds located on the scalp, trunk and hands was highly reliable and avoided suture removal [4–8]. In a randomized controlled trial, which compared Vicryl® Rapide with a non-absorbable suture for skin closure after open carpal tunnel release, authors described Vicryl® Rapide as a cost and time saving alternative to non-absorbable sutures [3]. In most of the publications Vicryl® Rapide has fallen out spontaneously within 2–3 weeks. Rates of postoperative adverse events for Vicryl® Rapide were reported as followed: wound infection 1–11% [2, 5, 6, 15], wound dehiscence 1–7% [6, 14], delayed wound healing 1–3.4% [4, 12], re-suturing of the wound 7.7% [6]. Suture removal, allergic reaction, inflammation and abscesses were rarely seen [1, 4, 5, 7, 9, 12–15]. Based on the amount of trials published, Vicryl® Rapide can be regarded as a market reference for absorbable braided suture material. The low rate of adverse events in our present study is comparable to the above described rates of adverse events of Vicryl® Rapide. Our small series indicates, that the use of Novosyn® Quick for skin closures in adults and paediatric patients is safe.

In order to evaluate the performance of Novosyn® Quick sutures all scars were assessed using the POSAS questionnaire 3 months after surgery. POSAS is a valid and reliable instrument with good internal consistency to evaluate scars (21-23) and appears to be the most comprehensive tool available for scar assessment [21–23]. In this study patients and observers rated the scars at 3 months follow-up close to normal skin. Results of the patient components of POSAS as well as the results of the overall opinions of the POSAS observer component were both in favour of the paediatric group. This may be attributed to the suture technique in the paediatric surgery centre, which nearly exclusively implemented a continuous intradermal method. The principal difference of scars in comparison to normal skin detected during follow-up assessment by observers and patients of both groups was the colour of the scar. At 3 months follow-up some scars in adult and paediatric patients showed a light red colour. In our observation this colour reflected a normal process of scar remodelling. A final evaluation of the scar colour at 3 months postoperatively may be too early due to ongoing remodelling of scar tissue. Overall our findings indicate that the use of Novosyn® Quick enables a very good to excellent aesthetic scar result both in adult and paediatric patients. The aesthetic outcome of wounds closed with Novosyn® Quick appears comparable with that of Vicryl® Rapide [1–3, 5, 7, 14, 15].

The intraoperative handling of the thread was assessed in four different categories. Visceral surgeons rated all categories with a level 4 (very good; range 4–4) in the adult group. Visceral surgeons used predominantly USP size 3/0 needle size 16 and rarely USP size 4/0 needle sizes 13. Thread ruptures did not occur among this suture sizes. Similarily, paediatric surgeons rated the suture handling characteristics in all categories in median with level 4 (range 1–5). A wider range of the median suture handling assessment by surgeons in the paediatric group may be attributed to the smaller suture size. Paediatric surgeons exclusively used USP size 5/0 with needle size 13. Among this group thread ruptures and knots in the thread handed out to the surgeon were accasionally found. Results of both groups indicate an overall very good handling of Novosyn® Quick for skin closure.

This study has some limitations. The follow-up period of 3 months was rather short and does not entirely reflect the remodelling of scar tissue, which continues for at least one year after surgery. The method of suturing was not standardised. Similarly, the assessments of cosmesis in the paediatric group were performed by a mix of parents and children. The pooling of these data involves the potential for confounding. Only one surgeon assessed the postoperative scar. Additional evaluators would have strengthened findings. However, all evaluators were experienced professionals and results were comparable with the results raised from patients and parents. Further weaknesses are the use of a historical control group, the small sample size and the limited variety of included ethnic skin types.

In our study, typical high volume cases of cutaneous sutures in adult and paediatric surgery were included. All patients were treated during daily clinical routine. The lengths of skin incisions reported in our study were corresponding to those reported in comparable literature and comprised short to medium long lengths of incisions. The centers in France and Germany have been selected, because they used the sutures on a routine basis. Authors believe that a fast-absorbing polyglactin or an equivalent suture type should be chosen as a first-line suture material for skin closure for wounds under low tension. Our results show, that, in consideration of the selected parameters, Novosyn® Quick can be regarded as safe.

## Conclusions

The SKINNOQ study is a single arm, prospective, observational study conducted in France and Germany involving adult and paediatric patients treated under daily clinical practice Novosyn*®* Quick sutures for skin closure. Novosyn*®* Quick sutures provided a very good intraoperative handling, were safe and allowed for excellent cosmetic results. Our findings indicate, that Novosyn*®* Quick is safe and can be regarded as a viable alternative to Vicryl*®* Rapide for skin closure in adult and paediatric patients.

## Additional file

Additional file 1: Dataset Description: Data 2. (XLSX 36 kb)

## Abbreviations

CRF: Case report form
Max: Maximum
Min: Minimum
POSAS: Patient-Oberserver-Scar-Assessment-Scale
SD: Standard deviation
SKINNOQ: Non-interventional, prospective study for SKIN closure to evaluate NOVOSYN® QUICK suture material
USP: United States Pharmacopeia
